# The relationship between Parkinson’s disease and gastrointestinal diseases

**DOI:** 10.3389/fnagi.2022.955919

**Published:** 2022-08-10

**Authors:** Jiaqi Zeng, Xinchan Wang, Fei Pan, Zhiqi Mao

**Affiliations:** ^1^Department of Gastroenterology and Hepatology, First Medical Center, Chinese PLA General Hospital, Beijing, China; ^2^Medical School of Chinese PLA, Beijing, China; ^3^Medical School of Nankai University, Tianjin, China; ^4^Department of Neurosurgery, First Medical Center, Chinese PLA General Hospital, Beijing, China

**Keywords:** Parkinson’s disease, gastrointestinal diseases, microbiota-gut-brain axis, gut microbiota, microbial metabolites, gut inflammation

## Abstract

An increasing number of studies have provided evidence for the hypothesis that the pathogenesis of Parkinson’s disease (PD) may derive from the gut. Firstly, Lewy pathology can be induced in the enteric nervous system (ENS) and be transported to the central nervous system (CNS) via the vagal nerve. Secondly, the altered composition of gut microbiota causes an imbalance between beneficial and deleterious microbial metabolites which interacts with the increased gut permeability and the gut inflammation as well as the systemic inflammation. The activated inflammatory status then affects the CNS and promotes the pathology of PD. Given the above-mentioned findings, researchers start to pay attention to the connection between PD and gastrointestinal diseases including irritable bowel syndrome, inflammatory bowel disease (IBD), microscopic colitis (MC), gastrointestinal infections, gastrointestinal neoplasms, and colonic diverticular disease (CDD). This review focuses on the association between PD and gastrointestinal diseases as well as the pathogenesis of PD from the gut.

## Introduction

Parkinson’s disease (PD) is the second most prevalent progressive neurodegenerative disease after Alzheimer’s disease. The incidence of PD, rising with age, is the highest in people aged around 80 (Ascherio and Schwarzschild, 2016). PD has the classical motor symptoms of rest tremor, muscular rigidity, bradykinesia, and postural and gait impairment (Cacabelos, 2017). Non-motor symptoms including impaired olfaction, constipation, depression, excessive daytime sleepiness, and rapid eye movement sleep behavior disorder (RBD) usually occur in the prodromal period of PD, before the onset of the primary motor symptoms. In this period, key features of PD pathology can already be identified (Klingelhoefer and Reichmann, 2015). The pathological hallmarks of PD are dopaminergic neurons loss in the substantia nigra pars compacta (SNpc) along with other parts of the brain, and the formation of Lewy bodies, mainly composed of an abnormally folded protein called α-synuclein, in the neurons of the central nervous system (CNS) and peripheral nervous system such as the enteric nervous system (ENS). Lewy pathology is hypothesized to develop following 6 stages. In the early stages before the onset of motor symptoms, Lewy bodies can be found in the peripheral nervous system, related to the non-motor symptoms. Moreover, pathological forms of α-synuclein aggregates besides Lewy bodies are also harmful to neurons (Kalia and Lang, 2015). Neuroinflammation also plays an important role in the pathology of PD. Reactive gliosis caused by activated astrocytes and microgliosis resulting from microglial activation is identified in the areas of neurodegeneration in PD (Phani et al., 2012). There is an increasing number of studies supporting that the above-mentioned pathology in the brain originates from the gastrointestinal tract. This article aims to review the roles the gut plays in the pathogenesis of PD and the association between PD and gastrointestinal diseases as well as the possible underlying mechanisms to reveal new insights into the etiology and pathophysiology of PD, explore early peripheral diagnosis of PD for early intervention, and facilitate timely identification and management of gastrointestinal comorbidities.

## Search strategy

We searched PubMed and Embase for articles published in English from Jan 1, 2000, to Jan 31, 2022 using subject words combined with free words. The search terms “parkinson,” “PD,” “gut microbiota,” “gastrointestinal diseases” were used. We also manually searched for additional relevant studies via the reference lists of included articles and review articles.

## The gut-derived pathogenesis of Parkinson’s disease

In recent years, the interaction between the gut, gut microbiota, and the brain has been demonstrated and named the microbiota-gut-brain axis (Cryan and Dinan, 2012). By the microbiota-gut-brain axis, gut microbiota can modulate the bidirectional gut-brain signaling pathways by interaction with the host (Cryan et al., 2019).

### Lewy pathology in enteric nervous system

The Lewy pathology has been identified in the gastrointestinal tract on an average of 7 years before the onset of the motor symptoms (Stokholm et al., 2016). BRAAK in 2006 put forward a hypothesis that the pathology of PD may be initiated from the gut and then the pathological substances transported from the gut to the brain. Subsequently, several studies presented evidence to support this hypothesis. Animal studies demonstrated that the intragastrically administered rotenone, a PD inducer, could cause pathological α-synuclein formation in ENS, in the vagal nerve, and then the brainstem. When vagotomy was performed, the pathological α-synuclein stopped transporting to the brain (Phillips et al., 2008; Kim et al., 2019). Furthermore, human α-synuclein injected into the intestinal wall of healthy mice could transport via the vagal nerve and reach the brainstem (Holmqvist et al., 2014). Interestingly, cohort studies found that compared to controls, patients receiving truncal vagotomy had a decreased risk of PD (Svensson et al., 2015; Liu et al., 2017); vagotomy referring to resecting the vagal nerve, is a treatment for peptic ulcers. The above-mentioned studies provide evidence for the hypothesis that Lewy pathology may be initiated in ENS and move toward the SNpc and other parts of the CNS (Braak et al., 2006).

### Gut microbiota

Gut microbiota is composed of a variety of microorganisms including bacteria, archaea, fungi, and viruses in the gastrointestinal tract. Studies presented heterogeneous or conflicting results of changes in the gut microbiota of PD patients and healthy controls. Nevertheless, taking 21 studies together, we summarized the gut microbiota with altered abundance in the PD group compared to the healthy control group in Table 1 (Scheperjans et al., 2015; Kuai et al., 2021; Vascellari et al., 2021; Yan et al., 2021; Zheng et al., 2021). The heterogeneity of results may be due to different sequencing methods of gut microbiota and confounding factors such as diets, age, and comorbidity. Despite the heterogeneity, these results provide candidates for further exploration of mechanisms.

**TABLE 1:** Altered abundance of gut microbiota in PD patients compared to controls.

	Bacteria	n↑*	n↓*
Phylum	Bacteroidetes		2
	Firmicutes	2	
Family	Prevotellaceae		7
	Lachnospiraceae		8
	Enterococcaceae	2	1
	Lactobacillaceae	6	2
	Blautia		3
	Akkermansia	7	
	Enterobacteriaceae	3	
Genus	Faecalibacterium		4
	Bacteroides	1	3
	Prevotella	1	7
	Clostridium	1	3
	Verrucomicrobiacea Faecalibacteriume	4	1
	Ruminococcus		2
	Lactobacillus	4	2
	Roseburia		3
	Oscillospira	4	
	Bifidobacterium	5	

*n↑ refers to number of studies reporting increased abundance of gut microbiota in PD patients compared to controls. n↓ refers to number of studies reporting decreased abundance.

The above-mentioned studies suggested a possible role of the altered composition of microbiota in the pathogenesis of PD, and a more direct relationship should be established by intervention studies. In a validated mouse model of PD (Sampson et al., 2016), antibiotic treatment alleviated, while microbial re-colonization promoted, the pathophysiology of PD in mice. Furthermore, mice colonized with fecal microbiota from PD patients had worse motor performance than that colonized with fecal microbiota from healthy controls. It indicates that the altered composition of microbiota represents a risk factor for PD.

The altered composition of microbiota in PD causes increased gut permeability and systemic inflammation via the translocation of bacteria and proinflammatory microbial metabolites (Forsyth et al., 2011; Fang, 2016; Felice et al., 2016). For example, a reduced Provotella population can result in decreased levels of protective short-chain fatty acids (SCFAs) and vitamins, such as thiamine and folate; moreover, Provotella can produce hydrogen sulfide which has a protective effect on dopaminergic neurons (Cakmak, 2015). The decreased SCFAs–producing Roseburia and Faecalibacterium may also facilitate neuroinflammation in PD. Akkermansia has been reported to have the ability to degrade the mucus layer of the gut. Then the increased gut permeability exposes the intestinal neural plexus to oxidative stress and/or pesticide/herbicide, causing abnormal aggregation of α-synuclein fibrils in the intestine (Nishiwaki et al., 2020). The abundances of Verrucomicrobia and Bacteroides were associated with elevated plasma levels of TNF-α and IFN-γ in PD patients, suggesting the role of Verrucomicrobia and Bacteroides in systemic inflammation (Lin et al., 2019). Besides, gut microbiota acts as an activator of immune cells in the brain, including brain-resident microglia (Erny et al., 2015). Activated microglia can release reactive oxygen, nitrogen species, and pro-inflammatory cytokines to induce neuroinflammation (Phani et al., 2012). Moreover, the α-synuclein-dependent motor dysfunction and gastrointestinal function could be significantly improved in antibiotics-treated animals (Sampson et al., 2016), suggesting the critical role of gut microbiota in the pathogenesis of PD. A preliminary study (Xue et al., 2020) including 15 PD patients reported relieved motor and non-motor symptoms after fecal microbiota transplantation (FMT), and compared with nasointestinal FMT, colonic FMT appears to be more effective. However, the follow-up period was 3 months, unable to evaluate the long-term effect of FMT on PD and it was a small sample-size self-controlled study that lacked a rigorous design. Hence, more randomized controlled trials are needed to further evaluate the efficacy and safety of FMT in treating PD.

### Microbial metabolites

It has been widely accepted that gut microbiota and their metabolites play a pivotal role in the pathogenesis of PD. The gut microbiota may contribute to the neurodegeneration of PD via the cumulative effects of microbial metabolites throughout the whole disease course before the onset of PD. The microbial metabolites in the gut can affect CNS via endocrine and neural pathways. In turn, the CNS alters gut movement, gut microbiota composition, secretion, permeability, and inflammation via autonomic interaction with the ENS (Zheng et al., 2021). SCFAs, including acetate, propionate, and butyrate are putative neuroprotective microbial metabolites by fermentation of fibers. Studies found that the levels of both serum and fecal SCFAs were differentially abundant between patients with PD and controls and the levels of SCFAs were significantly lower in PD patients (Unger et al., 2016; Russo et al., 2018; Wu et al., 2022). Additionally, butyrate was found to have a negative correlation with postural instability-gait disorder score and constipation severity (Tan et al., 2021). Bacteria-derived butyrate modulates hypoxia-inducible factor (HIF), which coordinates gut barrier protection (Kelly et al., 2015). The impaired gut barrier and gut inflammation have been suggested to be involved in the pathogenesis of PD, which will be discussed in the next section. However, an animal study demonstrated that SCFAs alone could activate microglia, induce increased α-synuclein aggregation in the brain and aggravate motor deficits (Sampson et al., 2016) by yet unknown pathways. For this, clinical studies are needed to further explore whether there is a positive relationship between PD and elevated SCFAs.

In addition to SCFAs, gut microbiota can also modulate the metabolism of amino acids. The levels of fecal branched-chain amino acids and aromatic amino acids were decreased in PD patients. Such amino acids were associated with an increased abundance of Alistipes, Rikenellaceae_RC9_gut_group, Bifidobacterium, Parabacteroides, and a decreased abundance of Faecalibacterium (Yan et al., 2021). Compared to healthy controls, the analysis of fecal metabolites showed higher levels of cadaverine, ethanolamine, hydroxypropionic acid, isoleucine and leucine, and phenylalanine. In contrast, glutamic acid, pyroglutamic acid, and succinic acid were significantly decreased in PD patients (Vascellari et al., 2020). The increased molecules may promote inflammatory responses and α-synuclein aggregation in the ENS via inducing oxidative stress and binding the N-terminal region of the amyloid beta peptide (Vascellari et al., 2021). Another study also revealed increased production of deleterious amino acids of protein degradation by gut microbiota, including p-cresol and phenylacetylglutamine in PD patients (Cirstea et al., 2020). The toxic amino acids have a harmful effect on the colonocytes (Diether and Willing, 2019) and may contribute to the disorder in the gut microenvironment.

Furthermore, gut microbiota plays a key role in metabolizing dietary constituents such as choline or L-carnitine into Trimethylamine N-oxide (TMAO), which is detectable in the cerebrospinal fluid. A study demonstrated that compared to healthy controls, plasma TMAO levels in patients with drug-naïve early stage PD were lower. Lower TMAO levels were related to more rapid longitudinal increases in doses of dopamine-based medications and higher risks for dementia (Chung et al., 2021). Another study also revealed reduced fecal levels of TMAO in PD, and fecal choline levels were higher in patients with motor response complications (occurring in the advanced stage of PD) (Tan et al., 2021). Studies have shown the protective effects of TMAO. TMAO could transform α-synuclein from one conformation to another, which may prevent the α-synuclein aggregation and formation of insoluble fibrils leading to PD (Jamal et al., 2017). When TMAO is at a high concentration level, α-synuclein forms oligomers, probably representing the physiologically folded form of the protein (Uversky et al., 2001). However, other studies reported a positive correlation between levels of TMAO and poor prognosis of various diseases, including neurological disorders and non-neurological disorders (Chung et al., 2021). TMAO has been reported to accelerate Aβ aggregation of Alzheimer’s disease and has been suggested to disrupt the blood-brain barrier (BBB) by reducing the expression of tight junction proteins like claudin-5 and tight junction protein-1. One of the mechanisms underlying the harmful effects of TMAO is demonstrated to be inflammation (Janeiro et al., 2018). Another study in 2020 demonstrated that plasma TMAO levels were elevated in patients with PD and correlated with disease severity and motor symptom progression (Chen et al., 2020). Likewise, a study revealed that plasma TMAO levels of patients with PD (with an average disease course of 8.1 years) were higher than that of the controls, and the levels were even higher in patients with motor fluctuation (Sankowski et al., 2020). The conflicting results may arise from the fact that the subjects in these studies were on medication and had a longer course of disease. The function of the above-mentioned microbial metabolites is summarized in Table 2.

**TABLE 2:** Potential roles of microbial metabolites in PD.

Microbial metabolites	Function	Potential roles in PD	References
SCFAs	Modulate HIF	Coordinate gut barrier protection and regulate inflammatory responses	Kelly et al., 2015
	Activate microglia	Induce increased α-synuclein aggregation in the brain and aggravate motor deficits	Sampson et al., 2016
Deleterious amino acids	Induce oxidative stress, bind the N-terminal region of the amyloid beta peptide and damage colonocytes	Promote inflammatory responses and α-synuclein aggregation in the CNS, and damage the gut barrier	Diether and Willing, 2019; Vascellari et al., 2021
TMAO	Transform the conformation of α-synuclein	Prevent the α-synuclein aggregation	Jamal et al., 2017
	Reducing the expression of tight junction proteins and cause inflammation	Disrupt the blood-brain barrier	Janeiro et al., 2018

SCFAs, Short-chain fatty acids; HIF, Hypoxia-inducible factor; CNS, Central nervous system; TMAO, Trimethylamine N-oxide.

### Gut inflammation

**FIGURE 1:**
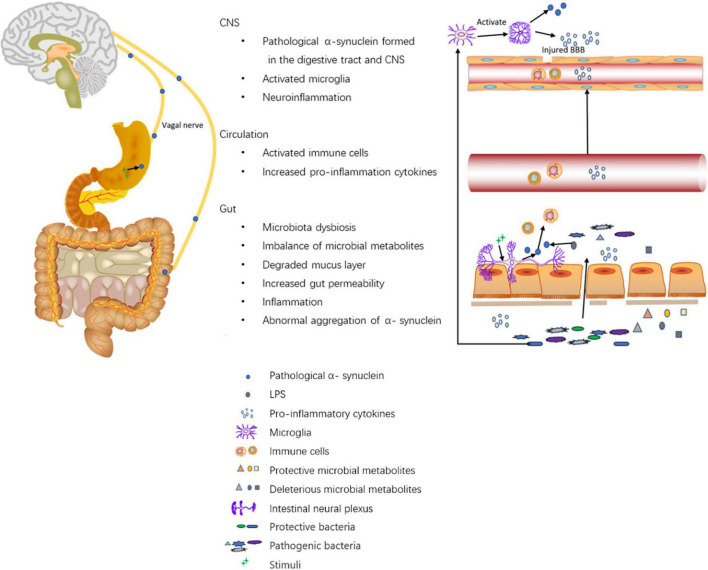
Roles of the digestive tract in the pathogenesis of PD. The altered composition of gut microbiota leads to altered levels of microbial metabolites such as SCFAs, vitamins, hydrogen sulfide, LPS, amino acid, and TMAO. First, on the one hand, SCFAs modulate gut barrier protection via HIF and modulate inflammation by inhibiting NF-kappaB activation; on the other hand, SCFAs alone may activate microglia and induce increased α-synuclein aggregation in the brain. Second, hydrogen sulfide has a protective effect on dopaminergic neurons. Third, LPS promotes intestinal α-synuclein aggregation. Forth, the imbalance of microbial metabolites can cause increased gut permeability and increased levels of inflammatory cytokines like TNF-α, IF-γ, IL-6, IL-1β, calprotectin, and NGAL. Moreover, Akkermansia can degrade the mucus layer of the gut. Then, the increased gut permeability exposes intestinal neural plexus to stimuli such as oxidative stress, pesticide/herbicide, traversed gut microbiota, and microbial metabolites, causing abnormal aggregation of α-synuclein fibrils in the intestine and inducing inflammation. Pathological α-synuclein promotes the activation of Th1 and Th17 in the circulation (accompanied by elevated levels of IL-6, TNF-α, IFN-γ, IL-1β, IL-2, IL-10, C-reactive protein, and RANTES), which activate microglia in the CNS through damaged BBB. Furthermore, gut microbiota dysbiosis can also activate microglia in the CNS. The activated microglia promote the aggregation of α-synuclein and releases reactive oxygen, nitrogen species, and pro-inflammatory cytokines to induce neuroinflammation. Additionally, Intra-gastric stimulation causes the formation of pathological α-synuclein within ENS, and the α-synuclein can directly be transmitted to the brain via the vagal trunk.

Gut inflammation has been suggested to be associated with PD. In a study with small sample size, compared with controls, biopsies from the ascending colon showed increased levels of pro-inflammatory cytokines including TNF-α, IF-γ, IL-6, and IL-1β (Devos et al., 2013). A Systematic review summarized the peripheral blood inflammatory cytokine data and concluded with higher levels of IL-6, tumor necrosis factor, IL-1β, IL-2, IL-10, C-reactive protein, and RANTES in patients with PD (Qin et al., 2016). Additionally, lipopolysaccharide (LPS), an inflammatory microbial metabolite, has been proven to accelerate α-synuclein aggregation. Some speculated that LPS initiated the pathology of PD in the gut via binding to α-synuclein (Bhattacharyya et al., 2019). Moreover, LPS is involved in the activation of inflammation in the colon and SN via the TLR4/MyD88/NF-κB signaling pathway (Zhao et al., 2021). Gastrointestinal inflammation may be responsible for increased intestinal permeability. Traversed gut microbiota and their metabolites then induce proinflammatory cytokine production in the ENS and aggregation of pathological α- synuclein. Pathological α-synuclein activates CD4^+^T-cells into Th1 and Th17. Th1 and Th17 then enter the brain via damaged BBB and activate microglia which induces neuroinflammation (Chen et al., 2019). Li et al. (2021) found that enriched Actinobacteria and Firmicutes phylum-associated epitopes were positively associated with inflammatory indicators such as neutrophil percentage, monocyte count/percentage, and white blood cell count in PD patients. Additionally, they were also positively associated with histidine degradation and proline biosynthesis pathways (Li et al., 2021). The relationship between gut microbiota, SCFAs, inflammation indicators, and the gut barrier in PD has been demonstrated. A study showed higher levels of calprotectin, lower levels of SCFA in stool, and lower levels of CXCL8 in plasma in PD patients than those of controls. The inflammatory markers, neutrophil gelatinase-associated lipocalin (NGAL) and calprotectin, and the gut permeability marker zonulin were also positively correlated. Stool SCFAs were negatively correlated with PD onset and symptom severity. PD patients had an increased abundance of Firmicutes and a decreased abundance of Prevotella. Prevotella is associated with higher levels of butyric acid and lower levels of NGAL and zonulin in stool. Moreover, the abundances of Butyricicoccus, Clostridium sensustricto, Roseburia, Lachnospiraceae, were positively correlated with levels of SCFAs, whereas the abundances of Enterobacteriaceae and Enterobacteriaceae, Akkermansia, Escherichia/Shigella, Flavonifractor, Intestinimonas, Phascolarctobacterium, and Sporobacter were negatively correlated with levels of SCFAs (Aho et al., 2021), in accordance with changes of gut microbiota in PD patients. In summary, the altered composition of gut microbiota may cause a decrease in protective SCFAs and gut inflammation. Gut inflammation may increase intestinal permeability, allowing the leakage of bacteria, and promoting the pathogenesis of PD. The mechanisms are summarized in Figure 1.

Based on the evidence illustrating the relationship between the gut and PD, several observational studies worldwide have started to focus on the association between gastrointestinal diseases and PD which is reviewed in the next part and collated in Table 3.

**TABLE 3:** The link between Parkinson’s disease and gastrointestinal diseases.

References	Diseases	Association	Possible mechanisms
Lai et al. (2014)	Irritable bowel syndrome	Positive	Abnormal gastrointestinal motility, small intestinal bacterial overgrowth
Liu et al. (2021)		Positive	
Mertsalmi et al. (2017a)		Positive	
Mishima et al. (2017)		Positive^#^	
Mertsalmi et al. (2017b)		Positive^#^	
Zhang et al. (2021)		Positive	
Lin et al. (2016)	Inflammatory bowel disease	Positive	Age, gene mutations, gut microbiota dysbiosis, intestinal inflammation
Peter et al. (2018)		Positive	
Weimers et al. (2019)		Positive	
Villumsen et al. (2019)		Positive	
Park et al. (2019)		Positive	
Camacho-Soto et al. (2018)		Negative	
Fujioka et al. (2017)		Insignificant^#^	
Kang et al. (2021)	Microscopic colitis	Positive*	Intestinal inflammation or drug side effects
		Insignificant	
Tian et al. (2021)	Gastrointestinal neoplasms	Positive	Gut microbiota dysbiosis, gene mutations
Lin et al. (2015) and Fang et al. (2021)		Negative* Positive*	
Macerollo et al. (2017)	Colonic diverticular disease	Positive	Abnormal gut motility, gut microbiota dysbiosis, intestinal inflammation

Positive refers to patients with gastrointestinal diseases having higher risks of PD. Negative refers to patients with gastrointestinal diseases having lower risks of PD. Positive^#^ refers to PD patients having a higher prevalence of gastrointestinal diseases. Positive* refers to PD patients having higher risks of gastrointestinal diseases. Negative* refers to PD patients having lower risks of gastrointestinal diseases. Insignificant refers to patients with gastrointestinal diseases not having higher risks of PD. Insignificant^#^ refers to PD patients not having a higher prevalence of gastrointestinal diseases.

## Gastrointestinal diseases and Parkinson’s disease

### Irritable bowel syndrome

IBS is a functional gastrointestinal disorder, characterized by chronic abdominal pain and altered bowel habits (including diarrhea, constipation, or alternating diarrhea and constipation). Several studies have revealed that patients with IBS had a higher risk of developing PD than those without IBS (Lai et al., 2014; Mertsalmi et al., 2017a; Liu et al., 2021; Zhang et al., 2021). Subsequently, a variety of studies were performed to investigate the prevalence of IBS in patients with PD and concluded that patients with PD had a higher prevalence of IBS than the general population (Mishima et al., 2017). Patients with IBS have an altered intestinal microenvironment and the subtypes of IBS may differ in the composition of gut microbiota. The subtypes of IBS include IBS with predominant constipation (IBS-C), IBS with predominant diarrhea (IBS-D), IBS with mixed bowel habits (IBS-M), and unclassified IBS. Another study in Finland further demonstrated that IBS-M was significantly more prevalent in PD patients than in healthy controls, while IBS-C and IBS-D were of no statistically significant difference from controls. And the fecal analysis showed a significantly lower abundance of the genus Prevotella and Bacteroides and the family Prevotellaceae in PD patients with IBS-like symptoms than that in PD patients without IBS-like symptoms (Mertsalmi et al., 2017b). Besides, abnormal gastrointestinal motility, including increased frequency and irregularity of luminal contractions, plays an important role in the pathogenesis of IBS. Increased gut motility can alleviate symptoms (Caldarella et al., 2002). Additionally, the link between ENS and CNS may also exist in the pathogenesis of IBS. Visceral hypersensitivity plays a role in IBS. The external stimuli are transported via receptors of the gut wall and afferent neural pathways toward the CNS, causing the perception of abdominal pain, gastrointestinal discomfort, and other symptoms. Furthermore, small intestinal bacterial overgrowth (SIBO) is frequently reported in IBS. SIBO is associated with an increase in the number and/or species of bacteria in the upper gastrointestinal tract. Of all, Methanobrevibacter smithii, contributing to a positive methane breath test, is associated with IBS-C. SIBO may lead to increased intestinal permeability, dysmotility, chronic inflammation, autoimmunity, decreased absorption of bile salts, and altered enteral and central neuronal activity in patients with IBS (Takakura and Pimentel, 2020). SIBO has also been reported to be prevalent in PD patients. And the eradication of SIBO in PD may improve motor dysfunctions. The underlying mechanisms may involve increased intestinal permeability, bacterial translocation, and systemic inflammation (Losurdo et al., 2020). Overall, IBS and PD may share the common multiple pathogenesis pathways as stated, thus linking to each other.

### Inflammatory bowel disease

Gastrointestinal inflammation has been suggested to be involved in the pathogenesis of PD. Microscopic colitis (MC) and inflammatory bowel disease (IBD), featuring the inflammation in the gut, may share a similar pathology. Thus, studies started to focus on the association between these diseases.

IBD consists of two major disorders: ulcerative colitis (UC) and Crohn’s disease (CD). UC affects the colon, while CD can affect the whole gastrointestinal tract from the mouth to the perianal area. The pathology and clinical characteristics of UC and CD overlap significantly with each other. Five cohort studies reported that IBD patients had an increased risk of developing PD (Lin et al., 2016; Peter et al., 2018; Park et al., 2019; Villumsen et al., 2019; Weimers et al., 2019). Of them, one study reported the risk was the highest among patients with CD (Lin et al., 2015), and another study reported that patients with UC had an increased risk of parkinsonism while patients with CD did not (Villumsen et al., 2019). However, 2 studies did not find a higher risk of PD in individuals with IBD. Besides, Camacho-Soto et al. (2018) showed that PD was inversely associated with IBD (OR = 0.85, 95% CI 0.80–0.91), both CD and UC. Another study also reported that in PD patients, no significant difference between the prevalence of CD and that of the general population (Fujioka et al., 2017). IBD and PD may share the following risk factors. First, the onset of IBD usually occurs in 2 age range. The second one is from 50 to 80 years old, which corresponds with the onset age of PD. Second, the leucine-rich repeat kinase 2 (LRRK2) gene is one of the major gene loci related to increased susceptibility for CD (Franke et al., 2010). LRRK2 mutations are also commonly observed in patients with PD (Tolosa et al., 2020). Third, although there is no consistent conclusion on the changes in gut microbiota in IBD patients, studies have reported an elevated abundance of Enterobacteriaceae, a decreased abundance of Lachnospiraceae, and SCFAs-producing bacteria Butyricicoccus in IBD patients, sharing a similar signature of altered microbiota with that of PD patients (Yang et al., 2021). Additionally, gut microbiota dysbiosis promotes the onset of IBD and the abundance of SCFAs-producing bacteria is reduced in IBD patients. Forth, intestinal inflammation is a hallmark of IBD. IBD and PD share the above-mentioned mechanisms of pathogenesis and this may account for the increased risk of PD in IBD patients.

Overall, these studies suggest a potential risk of PD in patients with IBD and indicate the effect of IBD treatment on the prevention of PD. The conflicting results may be due to some critical covariates not being considered, misclassification of IBD, and masked symptoms of PD. Moreover, there exist such limitations as ascertainment bias, biases related to confounder adjustments, and inaccessibility of data related to the severity of IBD and PD.

### Microscopic colitis

MC is a chronic inflammatory colonic disease, characterized by chronic, watery, non-bloody diarrhea. It typically affects middle-aged patients. It has been hypothesized that an impaired epithelial barrier may cause an increase in the transmucosal permeability of antigens and bacteria, resulting in immune dysregulation and intestinal inflammation (Andersen et al., 1993). A positive association between MC and IBD has been established. Patients with MC were reported to have a higher cc of IBD (Khalili et al., 2020). However, an assumed association between PD and MC does not exist. A Swedish cohort study showed that patients with MC did not have an increased risk of PD, but found that a previous diagnosis of PD was strongly associated with MC (Kang et al., 2021). Another study reported an increased risk of MC induced by levodopa/dopa-decarboxylase inhibitor (Ong et al., 2021). Therefore, the observed increased incidence of MC in PD patients may be due to the side effects of levodopa/dopa-decarboxylase inhibitor.

So far, no evidence has been presented to support a higher risk of PD in MC patients, and more studies should be conducted to explore it in the future.

### Gastrointestinal infections

Gastrointestinal infections (GIIs) can cause gut microbiota dysbiosis on the one hand, and gut inflammation on the other hand, which suggests a potential link between GIIs and PD. A prospective cohort study involving 228,485 individuals revealed an increased risk of PD in patients with GIIs compared with the control group with hazard ratio adjusted for multivariates. The pathogens included unspecified origin, viruses and bacteria (Nerius et al., 2020). There exists the question of whether there is a specific pathogen as a risk factor for PD. Population-based studies showed an increased risk of PD in chronic HP infection (Nielsen et al., 2012) and Clostridium difficile infection (CDI) (Kang et al., 2020). Interestingly, PD patients receiving Helicobacter pylori (HP) eradication therapy had significant clinical improvement in the symptoms of PD (Lolekha et al., 2021). However, whether it is due to HP eradication or gut microbiota restoration needs a further investigation in randomized controlled trials. The pathogens may exert effects via microbial products. Microbial products LPS could activate TLR4/MyD88/NF-κB signaling pathway in the SN and the colon. FMT administration could restore the gut microbial community and ameliorate the motor deficits in PD mice via the TLR4 signaling pathway (Zhao et al., 2021), suggesting a promising therapeutic target at the microbiota-gut-brain axis.

### Gastrointestinal neoplasms

A study using gene expression barcode algorithm and bioinformatics methods revealed that PD and gastric cancer have an association (Tian et al., 2021). This study indicates a positive association between PD and gastric cancer, but association does not equal causation. Therefore, clinical studies should be carried out to investigate the causal relationship. Despite the limitation, the underlying mechanisms of the association between PD and gastric cancer still deserve exploration. Gastric cancer is characterized by the aberrant expression of microRNAs (miRNAs). MiR-148a was down-regulated in gastric cancer tissues (Xia et al., 2014), corresponding with the finding that miR-148a expression levels were lower in PD patients than those in controls. MiR148a can affect cell proliferation, apoptosis, cell invasion, and metastasis. It is known to inhibit gastric cancer metastasis. Furthermore, miR148a-regulated proteins, such as ENAH (Isoform 2 of Protein enabled homolog), STX3 (Isoform B of Syntaxin-3), and TMED2 (Transmembrane emp24 domain-containing protein 2) have been reported to influence neurological development and function. Thus, decreased miR-148a expression may be the underlying link between PD and gastric cancer (Hu et al., 2013).

A systematic review including 17 studies concluded with a lower risk of colorectal cancer in PD patients in the Western population (Fang et al., 2021). In contrast, a cohort study in Taiwan reported a higher risk of developing subsequent cancers in PD patients, cancers including gastric cancer and colorectal cancer. Genetic, environmental, and dietary differences may account for the opposite results (Lin et al., 2015). Moreover, studies found that when ORs were adjusted for physician visits, odds of cancer in the total population after PD was reduce. The research by Freedman and Pfeiffer (2016) did not report ORs adjusted for physician visits, which may cause a bias. Nevertheless, we then discussed the possible mechanisms of these 2 conflicting results.

The lower risk of colorectal cancer in PD patients may result from altered microbiota composition and dysregulated molecular pathways. Dysfunction of the ubiquitin-proteasome system (UPS) can cause formation of the Lewy bodies, a pathological hallmark of PD (Sherman and Goldberg, 2001). While the function of UPS is usually up-regulated in CRC (Manasanch and Orlowski, 2017). Furthermore, studies revealed the activated PI3K/AKT/mTOR pathway in patients with CRC (Bahrami et al., 2018), which, however, may prevent the loss of dopaminergic neurons by inhibiting apoptosis, thus lowering the risk of PD (Leikas et al., 2017).

The higher risk of colorectal cancer in PD patients may be due to the following mechanisms. PD patients carrying the LRRK2 G2019S mutation have been reported to have an increased risk of colon cancer when compared with idiopathic PD patients (Agalliu et al., 2019). Moreover, PARK2 is a tumor suppressor gene, the mutation of which contributes significantly to the onset of PD. And PARK2 is inactivated and mutated in colon cancer (Veeriah et al., 2010). It indicates that genetic mutations in PD may be attributed to a higher risk of colorectal cancer. Though there is no uniform conclusion of CRC-associated microbiota structure, studies have reported enriched abundances of Streptococcus bovis, Enterotoxigenic Bacteroides fragilis, Fusobacterium nucleatum, Enterococcus faecalis, Escherichia coli, Peptostreptococcus anaerobius, and decreased abundances or depletion of butyrate-producing Clostridium butyicum and lactate-producing *S. thermophilus*. Studies also suggested the link between inflammation, genotoxins, oxidative stress, metabolites, biofilm underlying colorectal carcinogenesis, and gut microbiota (Veeriah et al., 2010). Hence, the altered microbiota may be attributed to the higher risk of colorectal cancer in PD.

The bidirectional association between gastrointestinal neoplasms and PD is in line with the hypothesis proposed in recent years that PD may be composed of two subtypes, “brain-first” and “body-first” types. Studies found that the brainstem-predominant type with stronger α-syn accumulation in the brainstem probably belonged to the “body-first” type, and a limbic/amygdala-predominant type, with stronger midbrain pathology, probably belonged to the “brain-first” type (Borghammer et al., 2021; Van Den Berge and Ulusoy, 2022). A recent study using multimodal imaging technique identified that compared to RBD-negative PD cases, the imaging data of RBD-positive PD patients and isolated RBD patients were characterized by initial loss of peripheral autonomic nervous system signal followed by loss of central signal, suggesting a “body-first” trajectory. By contrast, RBD-negative PD patient data showed an inverse order (Horsager et al., 2020). These findings support the two-subtype hypothesis of PD. Indeed, pathology in the gastrointestinal tract may be an initial cause for PD in some cases whereas a consequence of PD in other. Whether the subtype is related to the clinical type of PD and whether the different gut microbiome signatures can predict the subtype deserve further studies.

### Colonic diverticular disease

Diverticulosis is defined by the presence of diverticula, which refers to a sac-like protrusion of the colonic wall. A nationwide population-based cohort study revealed a significantly higher risk of PD in the colonic diverticular disease (CDD) cohort than in the control cohort (Macerollo et al., 2017). The underlying mechanisms of the link should be further explained. The pathological mechanisms of CDD remain to be elucidated, but altered gut microbiota is thought to be one of the mechanisms leading to CDD. Moreover, the abnormal gut motility results in an increase in the intraluminal pressure. Then, the intestinal mucosa and submucosa are probably more prone to herniation due to the increased intraluminal pressure (Tursi, 2016). The ENS plays a critical role in the regulation of gut motility (Sharma and Rao, 2017). A study found that patients with diverticular disease showed enteric neuropathy in the ENS characterized by myenteric and submucosal oligo-neuronal hypoganglionosis (Wedel et al., 2010). The pathology in the ENS suggests a possible link between CDD and PD. Future studies are needed to further detect pathological α-synuclein formation in the ENS of patients with CDD to confirm the association between PD and CDD. In addition, CDD can develop into diverticulitis, the macroscopic inflammation of diverticula which corresponds with the gut inflammation in PD. The above-mentioned underlying link between PD and CDD may explain the higher risk of PD in CDD patients.

## Conclusion

Since Braak et al. (2006) put forward the hypothesis that the pathogenesis of PD may start from the gut, accumulating evidence has been presented to support the hypothesis. Firstly, Lewy pathology can be induced in the ENS and transported to the CNS via the vagal nerve. Secondly, the altered composition of gut microbiota causes an imbalance between beneficial and deleterious microbial metabolites which interacts with the increased gut permeability and gut inflammation as well as systemic inflammation. The activated inflammatory status then affects the CNS and promotes the pathology of PD. Higher risks of subsequent PD were observed in IBS, IBD, GIIs, gastrointestinal neoplasms, and CCD. Although the underlying mechanisms of the link between PD and gastrointestinal diseases remain unclear, such gastrointestinal dysfunction or diseases may have common pathogenesis derived from the gut with PD as mentioned before. Interestingly, PD in turn could cause altered risks of subsequent gastrointestinal neoplasms, the bidirectional association suggested “brain-first” and “body-first” subtypes of PD. Previous studies focused on the prevalence of IBS, IBD, and CCD in PD patients, and whether PD is a risk factor for IBS, IBD, and CCD needs further investigation, which will play an important role in fully understanding the association between PD and gastrointestinal diseases. What is more, there exists the limitation that there are differences in ethnology, cultural background, and lifestyles that can cause heterogeneous results between studies. Hence, more studies from different nations and regions are needed to explore the relationship between PD and gastrointestinal diseases in the future. In conclusion, this review provides a new direction for pathophysiological research on PD, reasonable targets and grounds for peripheral diagnosis and treatment of early PD, as well as timely identification and management of gastrointestinal comorbidities.

## Author contributions

JZ discussed the contents and wrote the manuscript. XW searched for relevant studies and assessed the quality of them. FP and ZM reviewed the manuscript. All authors read and approved the final manuscript.
